# No evidence of flowering synchronization upon floral volatiles for a short lived annual plant species: revisiting an appealing hypothesis

**DOI:** 10.1186/s12898-019-0245-9

**Published:** 2019-08-07

**Authors:** Ute Fricke, Dani Lucas-Barbosa, Jacob C. Douma

**Affiliations:** 1 Centre for Crop Systems Analysis, Wageningen University, Droevendaalsesteeg 1, 6708 PB Wageningen, The Netherlands; 2 Laboratory of Entomology, Wageningen University, Droevendaalsesteeg 1, 6708 PB Wageningen, The Netherlands; 3 Present Address: Department of Animal Ecology and Tropical Biology, Biocentre, University of Würzburg, Würzburg, Germany; 4 Present Address: Laboratory of Bio-Communication & Ecology, ETH Zurich, Zurich, Switzerland

**Keywords:** Flowering synchronization, Flowering onset, Phenology, Plant–plant communication

## Abstract

**Background:**

Self-incompatible plants require simultaneous flowering mates for crosspollination and reproduction. Though the presence of flowering conspecifics and pollination agents are important for reproductive success, so far no cues that signal the flowering state of potential mates have been identified. Here, we empirically tested the hypothesis that plant floral volatiles induce flowering synchrony among self-incompatible conspecifics by acceleration of flowering and flower opening rate of non-flowering conspecifics. We exposed *Brassica rapa* Maarssen, a self-incompatible, in rather dense patches growing annual, to (1) flowering or non-flowering conspecifics or to (2) floral volatiles of conspecifics by isolating plants in separate containers with a directional airflow. In the latter, odors emitted by non-flowering conspecifics were used as control.

**Results:**

Date of first bud, duration of first flower bud, date of first flower, maximum number of open flowers and flower opening rate were not affected by the presence of conspecific flowering neighbors nor by floral volatiles directly.

**Conclusions:**

This study presents a compelling approach to empirically test the role of flower synchronization by floral volatiles and challenges the premises that are underlying this hypothesis. We argue that the life history of the plant as well as its interaction with pollinators and insect herbivores, as well as the distance over which volatiles may serve as synchronization cue, set constraints on the fitness benefits of synchronized flowering which needs to be taken into account when testing the role of floral volatiles in synchronized flowering.

**Electronic supplementary material:**

The online version of this article (10.1186/s12898-019-0245-9) contains supplementary material, which is available to authorized users.

## Background

The transition from the vegetative to flowering stage is irreversible and its timing is crucial [1, 2]: environmental conditions should be favorable, and for obligate outcrossing species, flowering conspecifics and pollinating agents to transfer pollen need to be present in the environment. Thus, flowering synchronization is critical for outcrossing species.

The flowering phenology of a plant depends on the interplay of endogenous signals and exogenous signals [3]. The most important exogenous signals are temperature, light conditions (such as photoperiod) and water availability [4]. For example, prolonged drought can be associated with accelerated flowering [5]. Shade and light quality can both play a role in flowering onset accelerating and delaying flowering, depending on the plant species [3]. Furthermore, fungal, viral and bacterial pathogen infection as well as herbivore attack can alter flowering phenology [6]. Endogenous factors that play a role in flowering phenology are plant hormones, sugar levels and the genetic makeup of the plant [3]. Differences in the genetic makeup may lead to variation in the individual timing of flowering within a population [7], while exogenous biotic and abiotic signals and their interactions may constrain flowering time of populations [6].

The intraspecific variation in flowering onset can be large [8] and constrains pollen transfer between individuals. Given the need of pollen from conspecifics, cues that signal the presence of flowering mates might have established to tune flowering among mates [9, 10].

Floral volatiles are outstanding candidate cues to signal the presence of flowering mates, for a number of reasons. First, floral volatiles are known to affect the physiology of neighboring plants, e.g. by inhibiting root growth [11]. Second, plant volatiles can promote defenses and growth in neighboring plants [12–15]. Third, floral volatiles are often produced in higher quantity than leaf volatiles which makes them likely detectable by nearby conspecifics [16]. Finally, floral volatiles are a reliable cue exploited by pollinators to locate flowers [17–19]. Floral volatile emission is often highest when a flower is ready for pollination, which makes unfertilized flowers particularly attractive to pollinators [20, 21]. Unfertilized flowers are, therefore, more attractive to pollinating agents than fertilized flowers and indeed floral scent changes or levels off after fertilization [22, 23].

Whether cues of conspecifics directly affect flowering time has never been investigated, although there is some indirect evidence that supports this idea [10]. For instance, butterfly egg deposition on *Brassica nigra* sped up flowering [24] and seed production of herbivore-infested plants and its non-infested neighbors, whereas floral volatile composition was changed upon exposure to butterfly eggs and caterpillars [16, 25]. Thus, additional floral volatile-based mechanisms may underlie flowering phenology.

In this study, we explored for the first time whether floral volatiles can change flowering phenology of younger conspecifics, i.e. whether floral volatiles can affect the onset of flowering and the rate of flower opening. We revisited the hypothesis by [10] of flower synchronization and challenged its premise that it is benefical for vegetative plants to advance the onset of flowering and the flower opening rate in response to floral volatiles.

## Results

The traits related to flowering phenology days from sowing until stem elongation (E), days from sowing until the first flower bud (FB) and days from sowing until the first flower opening (FF, flowering time) were not affected by the exposure to flowering or non-flowering neighboring conspecifics (neighboring experiment). Stem elongation manifested on average 38.3 ± 5.1 (mean ± SD) days from sowing [ANOVA, F(1,41) = 0.214, *P *= 0.646]. The first flower bud appeared at about 42.1 ± 5.3 days from sowing [ANOVA, F(1,41) = 0.002, *P *= 0.967] and lasted for about 6.1 ± 1.1 days [ANOVA, F(1,38) = 1.629, *P *= 0.210]. The first flower opened at about 47.4 ± 4.7 days from sowing [ANOVA, F(1,38) = 1.385, *P *= 0.247]. As in the neighboring experiment, the traits related to flowering phenology measured in the two-cylinder experiment were similar irrespectively of the floral volatiles exposure [ANOVA, E: F(1,25) = 0.008, *P *= 0.931; FB: F(1,24) = 1.704, *P *= 0.204; duration of first flower bud (FD): F(1,24) = 2.544, *P *= 0.124; FF: F(1,24) = 0.415, *P *= 0.526]. Importantly, no interactions were found between the trial number and the tested traits, but the traits related to flowering phenology differed between trials in the neighboring experiment on average up to 8.5 days. In the two-cylinder experiment only FD and FF differed between trials by an average of up to 2.5 days. Most of the traits related to flowering phenology had slightly lower values in the two-cylinder experiment when compared with the neighboring experiment presented above.

**Fig. 1:**
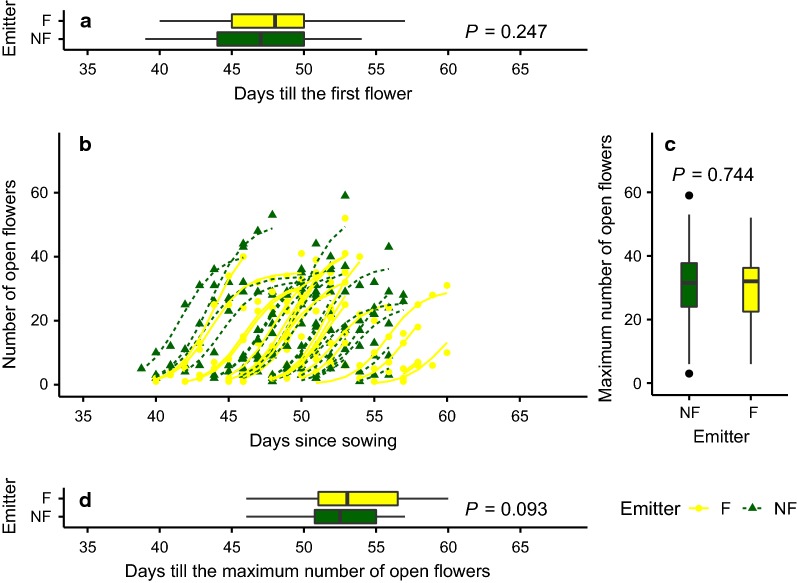
Relation between the number of open flowers and time when *Brassica rapa* plants were exposed to flowering (F; yellow) or non-flowering (NF; green) conspecific emitters in the neighboring experiment. **a** Maximum number of days until the first flower of *B. rapa*; **b** relationship between the number of open flowers and time until the maximum number of open flowers was reached for individual plants exposed to F or NF emitters modeled by a nonlinear mixed effects model based on the logistic function. Likelihood-ratio test showed no differences between Emitters; **c** maximum number of open flowers (NOF_max_); **d** number of days until the maximum number of open flowers was reached; Presented *P*-values for the type of emitter are based on a two-way ANOVA including the trial number at α = 0.05

When modeling the number of open flowers over time, the exposure to flowering or non-flowering (1) neighboring plants or (2) their odors only did not add explanatory value to the model [lr-test: (1) *P* = 0.627, (2) *P* = 0.818]. Both groups of *B. rapa* plants, exposed to flowering and non-flowering neighboring conspecifics, reached an equal maximum number of open flowers [*Asym*, (1) *P* = 0.687, (2) *P* = 0.468], started flowering at a similar time [*xmid*, (1) *P* = 0.226, (2) *P* = 0.639] and opened their flowers similarly quickly [*scal*, (1) *P* = 0.558, (2) *P* = 0.706] (Fig. 1; Additional file 1). However, the onset of flowering differed between the two subsequent trials in both experiments [*xmid*, (1) *P* < 0.001, (2) *P* = 0.007]. A model on the neighboring experiment data that included trial number in *scal* and the other parameters led to fitting problems, wherefore the covariate trial number was excluded from the explanation of *scal* in the neighboring experiment model. In case of the (2) two-cylinder experiment, these observations were made under consideration of a factor describing the number of plants elongated prior to exposure (preE). This factor was added as plants, which just had started to elongate, were used in the experiment due to a shortage of not yet elongated plants. A model with preE as explanatory factor was preferred (lr-test: *P* < 0.001). Plants showing elongated stems at the initiation of the experiment tended to reach a higher NOF_max_ than plants initiating stem elongation later (*P* = 0.074).

**Fig. 2:**
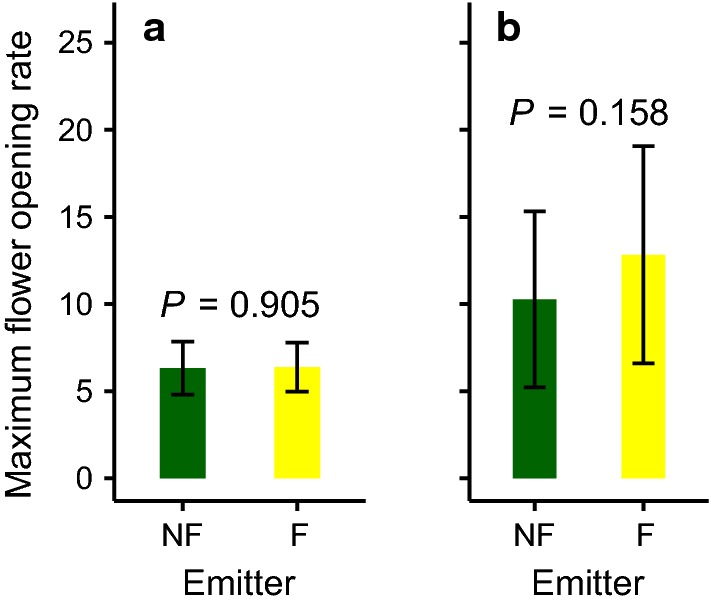
Maximum flower opening rates (mean ± SD) of *B. rapa* upon exposure to (odors of) flowering (F) and non-flowering (NF) conspecific emitters; **a** neighboring experiment; **b** two-cylinder experiment; *P*-values presented are based on the type of emitter in a two-way ANOVA with α = 0.05 including the trial number

Maximum flower opening rate (MFR) was similar between plants exposed to (odors of) flowering or non-flowering emitters in both experiments (Fig. 2). In the neighboring experiment MFR reached on average about 6.4 ± 1.4 (mean ± SD) flowers per day [ANOVA, F(1,37) = 0.014, *P* = 0.905], while in the two-cylinder experiment a maximal rate of average about 11.5 ± 5.7 flowers per day was reached [ANOVA, F(1,24) = 2.221, *P* = 0.149].

## Discussion

This study is, to the best of our knowledge, the first to evaluate the potential role of floral volatiles as cues to synchronize flowering among conspecifics. We designed two different types of experiments, one mimicking a field situation with flowering and non-flowering plants next to each other, and one in which plants were exposed to volatiles only, using a system of connected vessels, to test the effect of floral volatiles on conspecific neighboring plants of *B. rapa*. Flowering phenology of *B. rapa* was not affected by floral volatiles under the conditions tested.

Flowering synchronization may be achieved in different ways. Plants may shorten the time between the vegetative stage and bud formation; they may shorten the time between bud and flowering onset or increase the rate in which new flowers open. To date, it is not known if and when during the transition from the vegetative to the flowering stage a plant is responsive to volatile cues. Therefore, we measured a number of indices that could point to synchronization from as early as 8 days prior to the formation of the first bud till 12 days after flowering started. *Brassica rapa* needs a short period of time between bud formation and flower formation (4 days) which leaves little room for adjusting the flowering onset upon a volatile cue when the buds are already formed. Thus, any effect on synchronization would have likely been observed in the date of the first bud or the opening rate of the flowers. A power analysis showed that sample sizes of > 80 would have been needed to be able to show differences in day of first bud with 90% certainty at 0.05 significance level. Therefore, if any difference was detected, the fitness benefit of the earlier onset of flowering could be questioned.

Floral volatiles may be perceived by any organism present in the environment. Conspecific plants may use this cues and benefit of the fact that floral volatiles play a primary role in mediating interactions with pollinators. Volatile production is often highest prior to pollination, and the blend may change upon pollination [20, 23] and in this way plants maximize reproduction by guiding pollinators to flowers that have not been yet pollinated. In our experiment, exclusion of pollinators from the experiment prevented post-pollination effects and we assume we had maximized the availability of floral volatiles cues.

Our study questions the hypothesis of flower synchronization by volatile cues. This hypothesis is based on the premises that it is beneficial for conspecifics to flower simultaneously, and that floral volatiles provide reliable cues of the presence of flowering conspecifics plants. First, the benefit of synchronous flowering depends very much on other trophic levels [26, 27]. Synchronous flowering may result in more effective transfer of pollen by pollinators compared to asynchronous flowering plants [28], but too many flowering plants at the same time may lead to competition for pollinators such that asynchronous flowering is an advantage [26, 29, 30]. The same holds for florivores; synchronous flowering may decrease the probability of being eaten [28], but the opposite has also been recorded: asynchronous flowering plants are more likely to escape flower herbivores [31]. *B. rapa* is eaten by specialist herbivores, such as *Pieris rapae* and *Pieris brassicae*, who feed both on leaves and flowers. However, later instars prefer flowers over leaves despite the fact that flowers contain up to five times more glucosinolates than leaves [32, 33]. However, whether synchronous flowering is beneficial for *B. rapa* has not been studied.

Second, the payoff of synchronous flowering may as well depend on the life history of the plant. A study demonstrated that plants that produce a large number of flowers in a short time (mass flowering) showed highest synchrony [34]. Indeed, an offset of a few days in flowering in mass flowering plants will have a larger effect on pollination success compared to constant flowering plants. In addition, synchrony was observed in species that flowered in response to an unambiguous flowering cue (e.g. heavy rain), or where buds remained dormant until a specific cue became available [34]. For species that attain synchrony through responding to a specific environmental cue, it is not clear what information value can be added by floral volatiles, and whether there will be selection for flower synchronization by floral volatiles. Furthermore, one could argue that a minimal flower duration of the emitter plant is needed to be able to induce flowering of conspecifics by its volatiles and exchange pollen. *B. rapa* [35] flowers for ~ 30–40 days with the majority of the flowers produced in ~ 20 days and substantial variation across individuals [8], which should give a window of opportunity for synchronization. Alternatively, synchronization by volatiles may be not needed as the chance that the flowering schedules of two individuals will overlap is large. Thus, for this species, crosspollination may be assured by flowering over a relatively long period [25] and responding to floral volatiles may only become critical for this species to ensure reproduction in the presence of herbivores.

Third, adjusting the flowering time to an earlier flowering plant may imply that a plant should flower before it is optimal in terms of accumulated leaf biomass to maximize seed production [36]. Hence, earlier flowering may be beneficial in terms of increased pollination success, but may be suboptimal in terms of the amount of biomass accumulated and the maximum seed production attainable, unless plants are subjected to stress with the risk of missing out on an opportunity to reproduce. The cost of flowering earlier probably limits the extent to which plants synchronize or invest in synchronization.

Finally, even though there is substantial evidence that floral volatiles provide reliable cues of flowering and that pollinators can use olfactory cues to locate host plants [37, 38], evidences, however, showing that the distance over which floral volatiles disperse in meaningful concentrations for conspecific plants is limited, and may depend on the environmental conditions, such as ozone, wind speed and other canopy conditions [39, 40]. Plant volatiles as induced upon herbivory have been shown to induce a response in neighboring plants at distances up to 60 cm in bushy vegetation [41]. Even though floral volatiles are often emitted in higher amounts compared to volatiles emitted from leaves [16], its ability to serve as synchronization cue does probably not extend to the patch level. However, from a gene flow perspective, synchronization between patches is probably more important than within patches [25, 42].

Thus, the life history of the plant as well as its interaction with pollinators and insect herbivores, and the distance over which volatiles may serve as synchronization cue may set constraints on the fitness benefits of flowering in synchrony. This raises the question which species likely synchronize upon floral volatiles. We suggest to use representatives of the bromeliads, as they flower once in a lifespan and die after sexual reproduction [43] and, exceptionally, flowering can be induced through ethylene exposure [44, 45]. Alternatively, a stress-induced change in floral volatile composition, such as, upon plant exposure to herbivore attack may induce flowering both in the attacked plant as well as its surrounding conspecifics, such that reproduction is guaranteed before florivory takes place [25].

## Conclusions

Results of this study showed that floral volatiles did not accelerate floral transition or flowering and did not affect the flower opening rate of neighboring 2-week younger conspecific *B. rapa* plants. This study revisits the hypothesis of flower synchronization by floral volatiles by challenging the premises that are underlying this hypothesis. We argue that the role of floral volatiles in attaining synchrony must be seen in the context of the benefits and constraints that affect flowering synchrony, in particular the life history of the plant and its interaction with mutualists and antagonists. Our study offers an approach to empirically test the role of flower synchronization by floral volatiles and we discuss when we expect synchronizing flowering to be advantageous, opening further research avenues.

## Methods

### Plant cultivation

*Brassica rapa* Maarssen is an insect-pollinated, obligate outcrossing annual (biannual) plant, which in nature grows as an early successional plant in high density patches [Lucas-Barbosa D, personal observation]. Furthermore, the flowering phenology and floral volatiles of *B. rapa* are relatively well studied as well as its flowering traits [8, 33, 46, 47]. *B. rapa* seeds were obtained from a self-incompatible wild accession (following national guidelines for seed collection and storage). These *B. rapa* seeds were sown in germination boxes with a mixture of potting soil and sand (1:1 v/v). To narrow down the genetic variance and to flatten differences in starting conditions of the plants, only seeds of 1.1 to 1.3 mm in diameter were used. Seeds were stratified at 5 °C for 4 days. One-week old seedlings were transplanted individually to 3-L pots (ϕ 17 cm) filled with potting soil. The greenhouse compartment was conditioned to 21 °C during the day and to 15 °C at night. The plants were watered when needed. Plantings were staggered over a period of 5 weeks with a weekly sowing to achieve continuously available vegetative and flowering plants for the experiment.

The first experiment was carried out in a separate greenhouse compartment conditioned to 20 °C during the day and to 16 °C at night. The second experiment was run in the same compartment as used for growing the plants prior to the experiment. To avoid floral volatile exposure of the receiver plants prior to the experiment, planned receiver plants were kept up-wind of the flowering plants with respect to the greenhouse ventilation and at least at 2.5 m distance. The smell of flowering *B. rapa* could not be perceived by the human nose at the positions, where receiver plants were grown, and we assume that receiver plants were not exposed to floral volatiles prior to the experiment or, if so, at lower dose than under natural conditions where *B. rapa* plants generally occur in dense patches.

### Set ups of the experiments

**Fig. 3:**
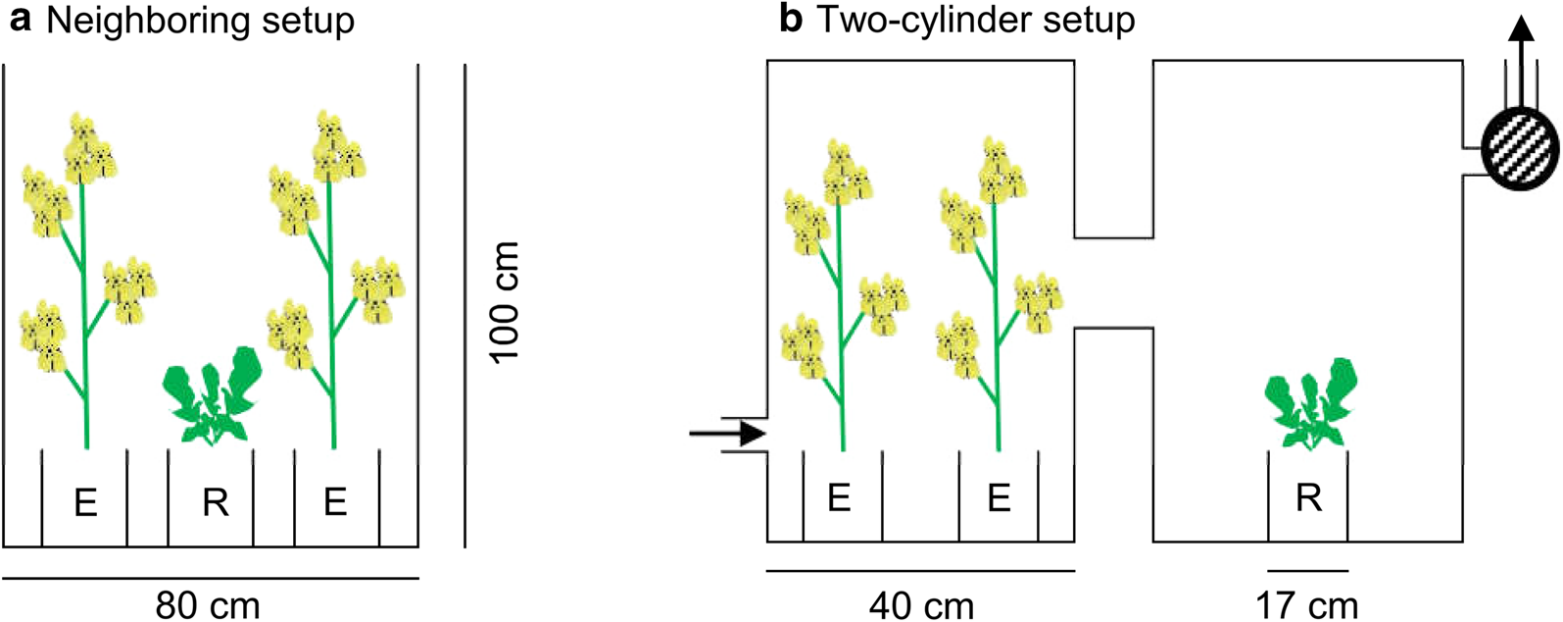
Layout of the two experiments used to test the impact of **a** flowering emitter plants (E) and **b** floral volatiles on a conspecific neighboring receiver plant (R). Non-flowering emitter plants and their odor were used as control

Two experiments were carried out to test whether (initially) vegetative *B. rapa* plants respond to flowering conspecific neighboring plants and floral volatiles of conspecifics. In the first experiment, plants were placed in proximity of flowering conspecific plants, hereafter called neighboring experiment (Fig. 3a). In the consecutive second experiment, plants were placed in separate containers with directional airflow (two-cylinder setup, Fig. 3b). Both experiments involved emitter and receiver plants. Emitter plants had at least two flower heads each, and in total at least 30 open flowers at the start of the exposure. The flowering emitter plants were on average 14 days older than conspecific receiver plants. Non-flowering conspecific neighboring plants, or their odors, served as the control. These non-flowering emitter plants included plants ranging from no stem elongation until branching and were replaced before they started flowering by another non-flowering plant. Receiver plants were in their vegetative stage and did not show stem elongation at the day plants were randomly selected for the experiment. The initial vegetative receiver plants and the non-flowering emitter plants were randomly selected from the available plants fulfilling the criteria 32 to 39 days from sowing.

#### (1) Neighboring setup

To investigate the impact of flowering plants on traits related to flowering phenology and the flower opening rate of neighboring conspecifics, two flowering or two non-flowering emitter plants were placed next to a vegetative receiver plant at a distance of 7 cm for a period of 21 days (Fig. 3a). The exposure of vegetative plants to other vegetative or non-flowering plants was used as control. Two flowering or two non-flowering (control) emitter plants were chosen to increase scent intensity and to mimic the patchy conditions in which they naturally occur. Each replication was enclosed by transparent foil to block horizontal air movement between different types of emitter and replicates. During the experiment, we made sure that the receiver and emitter plants were not touching each other and that the emitter plants were not overtopping the transparent enclosure. The experiment was repeated twice in time with 10 and 12 replicates per type of emitter, respectively.

#### (2) Two-cylinder setup

The aim of this experiment was to test the effect of floral volatiles on flower phenology of neighboring conspecifics. Equivalent to the neighboring set up experimental plants were exposed to odors of either two flowering plants or to two non-flowering plants. In the previous neighboring experiment, however, factors besides the floral volatiles, such as shading in the flowering emitter treatment might have confounded the flowering emitter effect. To mitigate side effects along the emitter effect, this two-cylinder, more controlled set up was used as follow up. In this case, the receiver plant and the two emitter plants were placed in two separate polyethylene cylinders (ϕ 42 cm, height: 1 m) connected through a tube (ϕ 10 cm, length ~ 3 cm) and a ventilation-sucking system (Fig. 3b). The cylinders were closed at the top with transparent foil forming a dome. The two-cylinder setup encompassed a total volume of about 1.5 m^3^. A ventilator blew air from the greenhouse compartment through the emitter container into the receiver container where the air was then sucked out. The ventilator was fixed in the inlet to provide overpressure in the connected vessels and make sure that air moved from the emitter to the receiver. The suction was set to about 400 mL min^−1^. The exposure was maintained for 19 days. Data was collected in two trials with seven replicates per type of emitter and trial. The setup was validated through volatile collection and subsequent GC–MS (Additional files 2, 3) of an empty receiver container, when two flowering plants were placed in the emitter container and when the whole system was empty. As positive control a dynamic headspace collection was done on two single flowering *B. rapa* plants.

### Measurements

Throughout the experiments, traits related to flowering phenology were measured on (focal) receiver plants, or were derived from these measurements, to get a complete picture of the hypothesized effect of floral volatiles on flowering phenology. The following traits were measured: The number of days (i) from sowing until stem elongation (E), (ii) until the first flower bud (FB) and (iii) until the first flower opening (FF, flowering time). From this, the duration of the first flower bud (FD) was calculated as the time difference between the first flower bud and the first flower opening. In addition, the number of open flowers (NOF) was counted on a daily basis to investigate the impact of floral volatiles on the flower opening rate. For this, a flower was considered open until the style became clearly longer than the anthers, and the petals started to wilt or bend from their orthogonal position. From NOF the maximum number of open flowers (NOF_max_) was derived per plant individually. In some cases, NOF_max_ was clearly reached, but in other cases only a relative maximum might have been reached as the NOF_max_ was limited by the observation period.

### Data analysis

The impact of flowering conspecific neighboring plants or floral volatiles on non-flowering conspecifics was tested by testing differences in a number of traits (E, FB, FF and FD). For these traits a two-way ANOVA was used with type of emitter and trial as factors.

Furthermore, a nonlinear mixed effects (nlme) model was used to fit a logistic relationship between the number of open flowers and time when exposed to flowering and non-flowering plants. The nonlinear mixed effect model allows to analyze the non-linear relationship in the data more precisely based on the multiple parameters, which makes it advantageous over a repeated measures ANOVA. A mixed effects model was taken as multiple observations were made on individual plants requiring the individual plant as random effect. The trial number was included as fixed effect. Observations made on NOF after plant individuals reached their maximum number of flowers were discarded to allow comparison between individuals. Because models allowing for count distributions (through so-called generalized nonlinear mixed effects models), are currently not implemented in commonly available statistics packages, the relationship between NOF and time was assumed to have a normal error distribution with a power variance structure (varPower) [48].

The logistic relationship between NOF and time (*x*) was modeled through three parameters:

1 $$f\left( x \right) = \frac{Asym}{{1 + e^{{\left( {xmid - x} \right)/scal}} }}$$

**Fig. 4:**
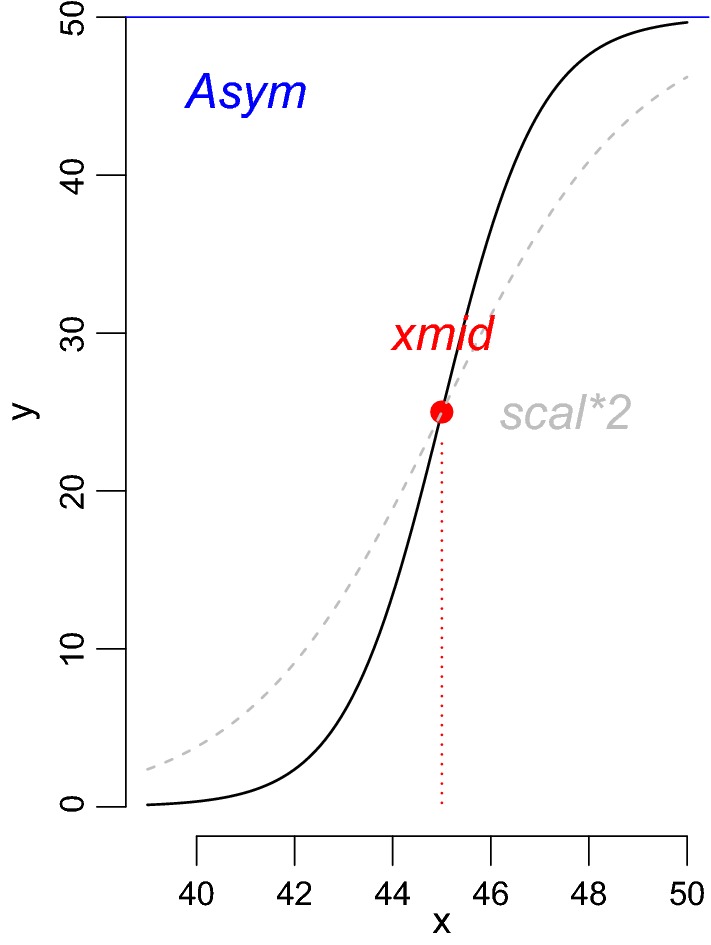
Describes the relationship between the number of open flowers and time that results in a sigmoid curve. The three parameters describe the upper asymptote (*Asym*, blue), the x-intercept of the inflection point (*xmid*, red) and the steepness (*scal*, grey) of the sigmoid curve

Upper asymptote (*Asym*), x-intercept of the inflection point (*xmid*) and steepness of the logistic curve (*scal*) (Eq. 1, Fig. 4). A higher value for the parameter *Asym* leads to a higher right-side asymptote. An increase of *xmid* is linked to a right shift of the entire curve on the x-axis. A lower *scal* causes a steeper curve, which is all else equal.

For both experiments, the covariates “type of emitter” and trial number were used to explain *Asym*, *xmid* and *scal*. In the two-cylinder experiment, an additional covariate was introduced correcting for the use of already elongated plants prior to the exposure (preE). The significance of the covariates was compared with a Likelihood ratio test (lr-test) at α = 0.05.

To assess the impact of floral volatiles on the maximum flower opening rate (MFR), the first derivative (Eq. 2) of the sigmoidal model (Eq. 1) was taken and evaluated under the assumption that all flowers had equal lifespan.

2 $$maximum \;flower\;opening\;rate : f^{\prime}\left( x \right) = \frac{Asym}{4 \cdot scal}$$

MFR was calculated for each plant by summing the fixed effects and random effects of the nlme models, which included the covariates type of emitter, trial number and additionally preE in the two-cylinder experiment as well as the individual plant as random effect. MFRs were tested on an emitter effect by a one-way ANOVA at α = 0.05.

For statistical analysis, the software R was used [49] with the packages nlme [50] and lmtest [51].

## Additional files

**Additional file 1.** Relation between the number of open flowers and time when *Brassica rapa* plants were exposed to odors of flowering or non-flowering conspecific emitters in the two-cylinder setup (Pendant to Fig. 1 for the two-cylinder setup). Relation between the number of open flowers and time when *Brassica rapa* plants were exposed to odors of flowering (F; yellow) or non-flowering (NF; green) conspecific emitters in the two-cylinder setup. a) Maximum number of days until the first flower of *B. rapa*; b) Relationship between the number of open flowers and time until the maximum number of flowers was reached for individual plants exposed to F or NF emitters modeled by a nonlinear mixed effects model based on the logistic function. Likelihood-ratio test showed no differences between Emitters; c) Maximum number of open flowers; d) Number of days until the maximum number of open flowers was reached; Presented *P*-values for the type of emitter are based on a two-way ANOVA including the trial number at α = 0.05.

**Additional file 2.** Volatile organic compounds collected in the headspace of *Brassica rapa*. Volatile organic compounds (VOCs) collected from the headspace of *Brassica rapa* (BR) (for details on the method see Bruinsma et al. [16]). The ratio of the peak areas in the gas chromatogram was calculated of VOCs collected from an empty receiver cylinder exposed to two flowering plants in the emitter cylinder (BR) and from an empty receiver cylinder when the emitter cylinder was left empty (BG). VOCs with a ratio of ≤ 0.8 (not grey) in a sample were determined by total ion composition (TIC) and retention index (RI). The sample number is indicated when the identity of a VOC was confirmed (conf.). VOCs with a BG/BR ratio ≤ 0.8, which were confirmed at least in one sample, are marked yellow. Confirmed VOCs by TIC in the *B. rapa* samples were additionally confirmed by squaring RI with literature and their peak areas in the gas chromatogram are presented by BR and BG in Additional file 3.

**Additional file 3.** Testing of the two-cylinder experiment - Peak areas of volatile organic compounds collected from an empty receiver cylinder exposed to odors of two flowering *Brassica rapa* and from an empty (emitter) cylinder. Peak areas of volatile organic compounds collected from an empty receiver cylinder exposed to odors of two flowering *Brassica rapa* (yellow) and from an empty (emitter) cylinder (background, grey), which showed a ratio of the peak areas for background/*B. rapa *≤ 0.8 and which identity was confirmed by squaring the retention index with literature. Values of peak area (median, 1st and 3rd quartiles, SD) obtained from total ion chromatogram. DF = 8 unless in c) DF = 7 as one *B. rapa* sample was excluded as outlier. *P*-values are based on an ANOVA at α = 0.05. Panel a) and e) present significantly higher amounts of volatiles in the *B. rapa* than in the background sample.

## Data Availability

The datasets used and/or analyzed during the current study are available from the corresponding author on reasonable request.

## Abbreviations

ANOVA: Analysis of Variance
E: days from sowing until stem elongation
F: flowering conspecifics
FB: days from sowing until first flower bud
FD: duration of first flower bud
FF: days from sowing until first flower opening
GC–MS: gas chromatography–mass spectrometry
MFR: maximum flower opening rate
NF: non-flowering conspecifics
NOF: number of open flowers
preE: elongated plants prior to start of exposure
SD: standard deviation

## References

[1] Huijser P, Schmid M (2011). The control of developmental phase transitions in plants. Development.

[2] Friedman J, Rubin MJ (2015). All in good time: understanding annual and perennial strategies in plants. Am J Bot.

[3] Srikanth A, Schmid M (2011). Regulation of flowering time: all roads lead to Rome. Cell Mol Life Sci.

[4] Bernier G, Havelange A, Houssa C, Petitjean A, Lejeune P (1993). Physiological signals that induce flowering. Plant Cell.

[5] Franks SJ (2011). Plasticity and evolution in drought avoidance and escape in the annual plant Brassica rapa. New Phytol.

[6] Kazan K, Lyons R (2016). The link between flowering time and stress tolerance. J Exp Bot.

[7] Fox GA (2003). Assortative mating and plant phenology: evolutionary and practical consequences. Evol Ecol Res..

[8] Weis AE (2005). Direct and indirect assortative mating: a multivariate approach to plant flowering schedules. J Evol Biol..

[9] Rivkin LR, Case AL, Caruso CM (2015). Frequency-dependent fitness in gynodioecious *Lobelia siphilitica*. Evolution.

[10] Caruso CM, Parachnowitsch AL (2016). Do plants eavesdrop on floral scent signals?. Trends Plant Sci.

[11] Horiuchi J-I, Badri DV, Kimball BA, Negre F, Dudareva N, Paschke MW, Vivanco JM (2007). The floral volatile, methyl benzoate, from snapdragon (*Antirrhinum majus*) triggers phytotoxic effects in *Arabidopsis thaliana*. Planta..

[12] Ninkovic V (2003). Volatile communication between barley plants affects biomass allocation. J Exp Bot.

[13] Peng J, van Loon JJA, Zheng S, Dicke M (2011). Herbivore-induced volatiles of cabbage (*Brassica oleracea*) prime defence responses in neighbouring intact plants. Plant Biol..

[14] Ueda H, Kikuta Y, Matsuda K (2012). Plant communication: mediated by individual or blended VOCs?. Plant Signal Behav.

[15] Karban R, Yang LH, Edwards KF (2014). Volatile communication between plants that affects herbivory: a meta-analysis. Ecol Lett.

[16] Bruinsma M, Lucas-Barbosa D, ten Broeke CJM, van Dam NM, van Beek TA, Dicke M, van Loon JJA (2014). Folivory affects composition of nectar, floral odor and modifies pollinator behavior. J Chem Ecol.

[17] Chittka L, Raine NE (2006). Recognition of flowers by pollinators. Curr Opin Plant Biol.

[18] Raguso RA (2008). Wake up and smell the roses: the ecology and evolution of floral scent. Annu Rev Ecol Evol S.

[19] Šimpraga M, Takabayashi J, Holopainen JK (2016). Language of plants: where is the word?. J Integr Plant Biol.

[20] Rodriguez-Saona C, Parra L, Quiroz A, Isaacs R (2011). Variation in highbush blueberry floral volatile profiles as a function of pollination status, cultivar, time of day and flower part: implications for flower visitation by bees. Ann Bot.

[21] Muhlemann JK, Klempien A, Dudareva N (2014). Floral volatiles: from biosynthesis to function. Plant Cell Environ.

[22] Negre F, Kish CM, Boatright J, Underwood B, Shibuya K, Wagner C, Clark DG, Dudareva N (2003). Regulation of methylbenzoate emission after pollination in snapdragon and petunia flowers. Plant Cell..

[23] Lucas-Barbosa D, Sun P, Hakman A, Beek TA, van Loon JJ, Dicke M (2016). Visual and odour cues: plant responses to pollination and herbivory affect the behaviour of flower visitors. Funct Ecol.

[24] Pashalidou FG, Lucas-Barbosa D, van Loon JJ, Dicke M, Fatouros NE (2013). Phenotypic plasticity of plant response to herbivore eggs: effects on resistance to caterpillars and plant development. Ecology.

[25] Lucas-Barbosa D, van Loon JJ, Gols R, Beek TA, Dicke M (2013). Reproductive escape: annual plant responds to butterfly eggs by accelerating seed production. Funct Ecol..

[26] Ims RA (1990). The ecology and evolution of reproductive synchrony. Trends Ecol Evol.

[27] Elzinga JA, Atlan A, Biere A, Gigord L, Weis AE, Bernasconi G (2007). Time after time: flowering phenology and biotic interactions. Trends Ecol Evol.

[28] Augspurger CK (1981). Reproductive synchrony of a tropical shrub: experimental studies on effects of pollinators and seed predators in *Hybanthus prunifolius* (Violaceae). Ecology.

[29] Gomez JM (1993). Phenotypic selection on flowering synchrony in a high mountain plant, *Hormathophylla spinosa* (Cruciferae). J Ecol.

[30] Parra-Tabla V, Vargas CF (2007). Flowering synchrony and floral display size affect pollination success in a deceit-pollinated tropical orchid. Acta Oecol..

[31] English-Loeb GM, Karban R (1992). Consequences of variation in flowering phenology for seed head herbivory and reproductive success in *Erigeron glaucus* (Compositae). Oecologia.

[32] Smallegange RC, van Loon JJA, Blatt SE, Harvey JA, Agerbirk N, Dicke M (2007). Flower vs. leaf feeding by pieris brassicae: glucosinolate-rich flower tissues are preferred and sustain higher growth rate. J Chem Ecol..

[33] Schiestl FP (2014). Correlation analyses between volatiles and glucosinolates show no evidence for chemical defense signaling in *Brassica rapa*. Front Ecol Evol..

[34] Augspurger CK (1983). Phenology, flowering synchrony, and fruit set of six neotropical shrubs. Biotropica.

[35] Austen EJ, Weis AE (2015). What drives selection on flowering time? An experimental manipulation of the inherent correlation between genotype and environment. Evolution.

[36] Vermeulen PJ (2015). On selection for flowering time plasticity in response to density. New Phytol.

[37] Knudsen JT, Tollsten L (1993). Trends in floral scent chemistry in pollination syndromes: floral scent composition in moth-pollinated taxa. Bot J Linn Soc.

[38] Dudareva N, Pichersky E, Gershenzon J (2004). Biochemistry of plant volatiles 1. Plant physiol..

[39] McFrederick QS, Kathilankal JC, Fuentes JD (2008). Air pollution modifies floral scent trails. Atmos Environ.

[40] Aartsma Y, Bianchi FJJA, Werf W, Poelman EH, Dicke M (2017). Herbivore-induced plant volatiles and tritrophic interactions across spatial scales. New Phytol.

[41] Karban R, Shiojiri K, Huntzinger M, McCall AC (2006). Damage-induced resistance in sagebrush: volatiles are key to intra- and interplant communication. Ecology.

[42] Rasmussen IR, Brødsgaard B (1992). Gene flow inferred from seed dispersal and pollinator behaviour compared to DNA analysis of restriction site variation in a patchy population of *Lotus corniculatus* L. Oecologia.

[43] Amasino R (2009). Floral induction and monocarpic versuspolycarpic life histories. Genome Biol.

[44] Espinosa MEÁ, Moreira RO, Lima AA, Ságio SA, Barreto HG, Luiz SLP, Abreu CEA, Yanes-Paz E, Ruíz YC, González-Olmedo JL (2017). Early histological, hormonal, and molecular changes during pineapple (*Ananas comosus* (L.) Merrill) artificial flowering induction. J Plant Physiol..

[45] Iqbal N, Khan NA, Ferrante A, Trivellini A, Francini A, Khan MIR (2017). Ethylene role in plant growth, development and senescence: interaction with other phytohormones. Front Plant Sci..

[46] Weis AE, Kossler TM (2004). Genetic variation in flowering time induces phenological assortative mating: quantitative genetic methods applied to *Brassica rapa*. Am J Bot.

[47] Zu P, Blanckenhorn WU, Schiestl FP (2016). Heritability of floral volatiles and pleiotropic responses to artificial selection in *Brassica rapa*. New Phytol.

[48] Zuur AF, Ieno EN, Walker NJ, Saveliev AA, Smith GM, Gail M, Krickeberg K, Samet JM, Tsiatis A, Wong W (2009). Dealing with heterogeneity. Statistics for biology and health: mixed effects models and extensions in ecology with R.

[49] R Core Team. R: a language and environment for statistical computing. R version 3.4.1. Vienna, Austria: R Foundation for Statistical Computing; 2017. https://www.R-project.org/.

[50] Pinheiro J, Bates D, DebRoy S, Sarkar D, R Core Team. nlme: linear and nonlinear mixed effects models. R package version 3.1-131; 2017. https://CRAN.R-project.org/package=nlme.

[51] Hothorn T, Zeileis A, Farebrother RW, Cummins C. Lmtest: testing linear regression models. R package version 0.9-35; 2017. https://CRAN.R-project.org/package=lmtest.

