# Supplementary figures and images for: No evidence of flowering synchronization upon floral volatiles for a short lived annual plant species: revisiting an appealing hypothesis
Source: BMC Ecol. 2019 Aug 7;19:29. doi: 10.1186/s12898-019-0245-9 (PMC6685148; doi:10.1186/s12898-019-0245-9)

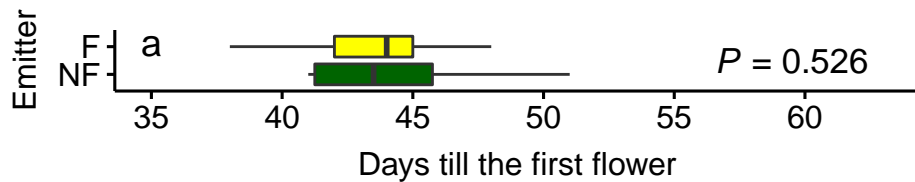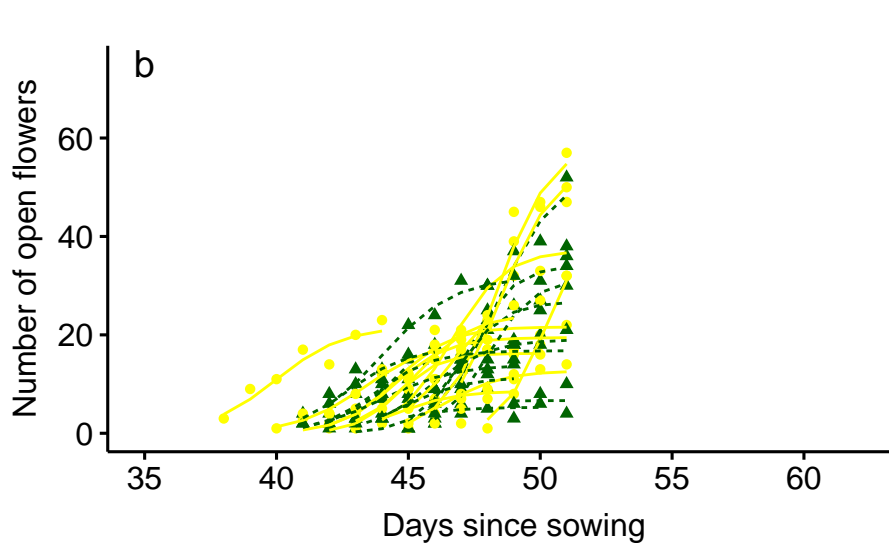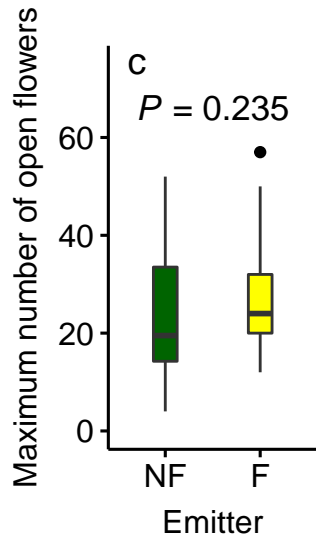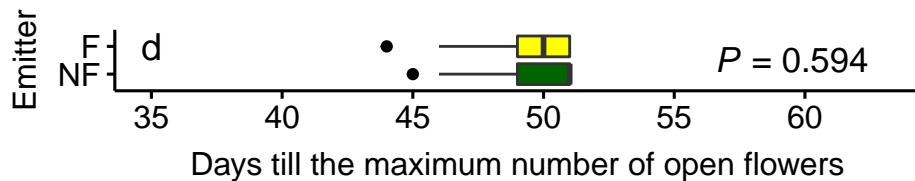

Emitter — F — NF

Supplement: Supplementary file 1 — Additional file 1. Relation between the number of open flowers and time when Brassica rapa plants were exposed to odors of flowering or non-flowering conspecific emitters in the two-cylinder setup (Pendant to Fig. 1 for the two-cylinder setup). Relation between the number of open flowers and time when Brassica rapa plants were exposed to odors of flowering (F; yellow) or non-flowering (NF; green) conspecific emitters in the two-cylinder setup. a) Maximum number of days until the first flower of B. rapa; b) Relationship between the number of open flowers and time until the maximum number of flowers was reached for individual plants exposed to F or NF emitters modeled by a nonlinear mixed effects model based on the logistic function. Likelihood-ratio test showed no differences between Emitters; c) Maximum number of open flowers; d) Number of days until the maximum number of open flowers was reached; Presented P-values for the type of emitter are based on a two-way ANOVA including the trial number at α = 0.05. [file 12898_2019_245_MOESM1_ESM.pdf]
