# Supplementary material for: No evidence of flowering synchronization upon floral volatiles for a short lived annual plant species: revisiting an appealing hypothesis
Source: BMC Ecol. 2019 Aug 7;19:29. doi: 10.1186/s12898-019-0245-9 (PMC6685148; doi:10.1186/s12898-019-0245-9)
Supplement: Supplementary file 2 — Additional file 2. Volatile organic compounds collected in the headspace of Brassica rapa. Volatile organic compounds (VOCs) collected from the headspace of Brassica rapa (BR) (for details on the method see Bruinsma et al. [16]). The ratio of the peak areas in the gas chromatogram was calculated of VOCs collected from an empty receiver cylinder exposed to two flowering plants in the emitter cylinder (BR) and from an empty receiver cylinder when the emitter cylinder was left empty (BG). VOCs with a ratio of ≤ 0.8 (not grey) in a sample were determined by total ion composition (TIC) and retention index (RI). The sample number is indicated when the identity of a VOC was confirmed (conf.). VOCs with a BG/BR ratio ≤ 0.8, which were confirmed at least in one sample, are marked yellow. Confirmed VOCs by TIC in the B. rapa samples were additionally confirmed by squaring RI with literature and their peak areas in the gas chromatogram are presented by BR and BG in Additional file 3. [file 12898_2019_245_MOESM2_ESM.pdf]

Additional file 2. Volatile organic compounds (VOCs) collected from the headspace of *Brassica rapa* Maarssen (BR) (for details on the method see Bruinsma *et al.* 2014). The ratio of the peak areas in the gas chromatogram was calculated of VOCs collected from an empty receiver cylinder exposed to two flowering plants in the emitter cylinder (BR) and from an empty receiver cylinder when the emitter cylinder was left empty (BG). VOCs with a ratio of ≤ 0.8 (not grey) in a sample were determined by total ion composition (TIC) and retention index (RI). The sample number is indicated when the identity of a VOC was confirmed (conf.). VOCs with a BG/BR ratio ≤ 0.8, which were confirmed at least in one sample, are marked yellow. Confirmed VOCs by TIC in the *B. rapa* samples were additionally confirmed by squaring RI with literature and their peak areas in the gas chromatogram are presented by BR and BG in Additional file 3.

**VOCs in headspace collection (BR)**	**Peak area of BG/BR≤0.8**	**RT**	**Conf. by TIC (BR)**	**Conf. by TIC (BG)**	**RI (cal.)**	**Conf. by RI (BR)**
2-Methylbutanenitrile	2, 3, 6	6.94	2, 3	4	723	**all**
3-Methyl-3-buten-1-ol	4, 6	7.12	no	-	729	-
Tiglic-aldehyde	1-6	7.48	no	-	742	-
Dimethyl disulfide	1-4, 6	7.6	1-4, 6	all	747	**all**
Methallyl cyanide	1	7.99	1	all	761	**all**
2-Methylbutanoic acid, methyl ester	1, 3, 4, 6	8.38	no	-	775	-
2,4-Dithiapentane	1	11.95	no	-	898	-
Prenyl acetate	6	12.59	no	-	919	-
Anisole	6	12.67	no	-	922	-
Butane, 1-isothiocyanato	2, 3, 6	13.02	no	-	934	-
α-pinene		13.3	1-4, 6	all	943	**all**
Camphene		13.8	-	-	960	-
Dimethyl trisulfide	1-4, 6	14.43	no	-	982	-
3-Butenyl isothiocyanate	1-4, 6	14.51	1-4, 6	all	984	**all**
β-pinene	6	14.64	6	all	989	-
β-Myrcene		14.7	-	-	991	-
3-Hexen-1-ol, acetate, (Z)-	4	15.06	4	-	1003	**all**
Hexyl acetate	4	16.03	no	-	1038	-
Limonene		16.02	-	-	1038	-
β-Myrcene hydroperoxide	1-3, 6	16.34	no	-	1049	-
Phenylacetaldehyde	1-6	16.45	no	-	1053	-
Methyl benzoate	3, 6	17.84	no	-	1103	-
4,8-Dimethyl-1,3,7-nonatriene, (*E*)-		18.16	-	-	1116	-
Benzyl cyanide		19.03	-	-	1148	-
Limonene, 8,9-epoxy-		21.24	-	-	1233	-
β-Caryophyllene, (E)-	2, 3	26.26	no	-	1446	-
Geranylacetone		26.38	-	-	1452	-
α-Farnesene, (Z,E)-		27.29	-	-	1493	-
α-Farnesene, (E,E)-	1-3, 6	27.62	no	-	1508	-
1,3,7,11-Tridecatetraene, 4,8,12-trimethyl-, (E,E)-		29.06	-	-	1578	-
