# Supplementary material for: No evidence of flowering synchronization upon floral volatiles for a short lived annual plant species: revisiting an appealing hypothesis
Source: BMC Ecol. 2019 Aug 7;19:29. doi: 10.1186/s12898-019-0245-9 (PMC6685148; doi:10.1186/s12898-019-0245-9)
Supplement: Supplementary file 3 — Additional file 3. Testing of the two-cylinder experiment - Peak areas of volatile organic compounds collected from an empty receiver cylinder exposed to odors of two flowering Brassica rapa and from an empty (emitter) cylinder. Peak areas of volatile organic compounds collected from an empty receiver cylinder exposed to odors of two flowering Brassica rapa (yellow) and from an empty (emitter) cylinder (background, grey), which showed a ratio of the peak areas for background/B. rapa ≤ 0.8 and which identity was confirmed by squaring the retention index with literature. Values of peak area (median, 1st and 3rd quartiles, SD) obtained from total ion chromatogram. DF = 8 unless in c) DF = 7 as one B. rapa sample was excluded as outlier. P-values are based on an ANOVA at α = 0.05. Panel a) and e) present significantly higher amounts of volatiles in the B. rapa than in the background sample. [file 12898_2019_245_MOESM3_ESM.pdf]

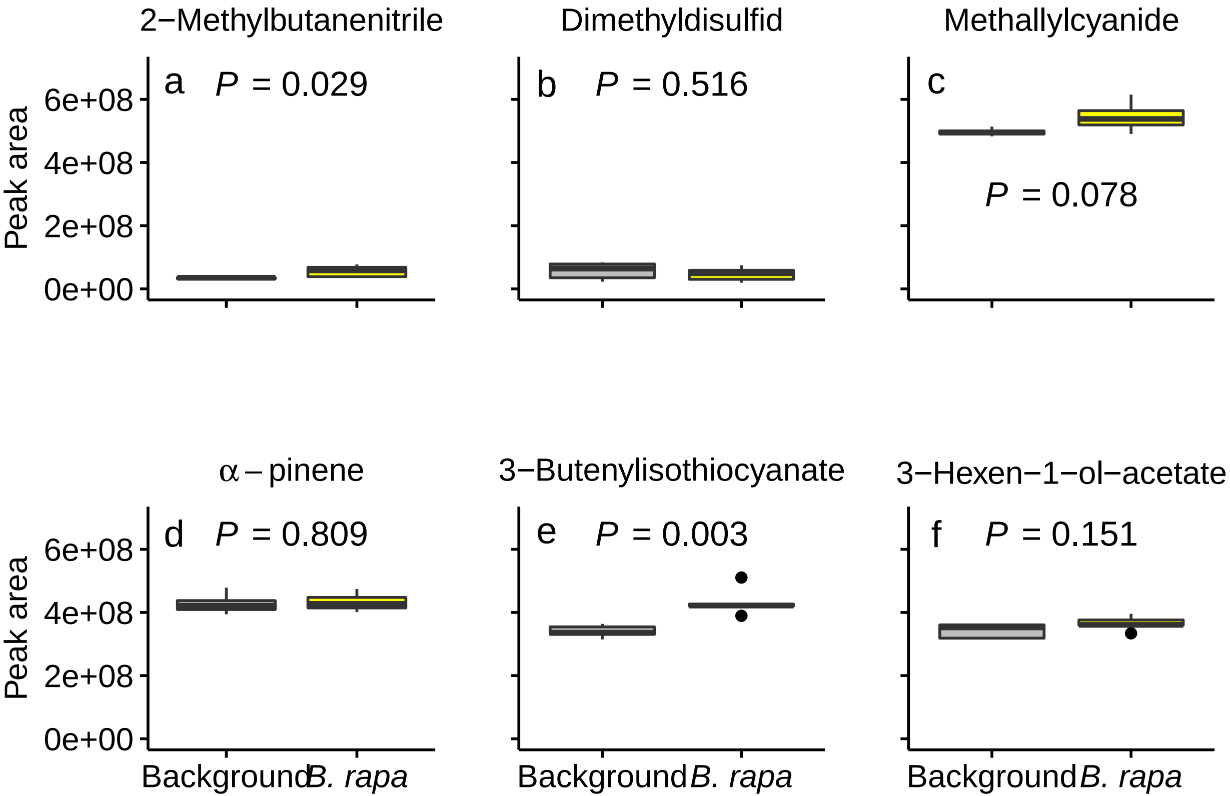

2−Methylbutanenitrile
Dimethyldisulfid
Methallylcyanide
a P = 0.029
b P = 0.516
c
P = 0.078
α − pinene
3−Butenylisothiocyanate
3−Hexen−1−ol−acetate
d P = 0.809
e P = 0.003
f P = 0.151
Peak area
6e+08
4e+08
2e+08
0e+00
Background
B. rapa
